# Osteoma of the Pharynx That Developed from the Hyoid Bone

**DOI:** 10.1155/2014/732096

**Published:** 2014-12-11

**Authors:** Akira Hagiwara, Noriko Nagai, Yasuo Ogawa, Mamoru Suzuki

**Affiliations:** ^1^Department of Otolaryngology, Tokyo Medical University, 6-7-1 Nishishinjuku, Shinjuku-ku, Tokyo 160-0023, Japan; ^2^Department of Otolaryngology, Kohsei Chuo General Hospital, 1-11-7 Mita, Meguro-ku, Tokyo 153-8581, Japan

## Abstract

This paper reports on apparently the first case of a pharyngeal osteoma that developed from the hyoid bone. An 84-year-old man's, presenting symptom was a slight throat pain. Endoscopic examination revealed a huge mass occluding the pharyngeal space. CT scan of the neck showed a large osseous mass adjacent to the hyoid bone. Transoral resection with tracheostomy was performed. Histopathologically, the tumor consisted of mature lamellar bone without a fibrous component. For two years postoperatively, the patient has been free from throat symptoms and signs of recurrence. Osteomas are benign, slow-growing tumors. They rarely develop symptoms or cause functional disturbance. We performed total resection to avoid further functional disturbance as the osteoma was huge. To the best of our knowledge, this is the first report on an osteoma that occupied the pharyngeal space and developed from the hyoid bone.

## 1. Introduction

Osteoma is a benign osteogenic lesion characterized by the proliferation of mature cancellous or compact bone [1, 2]. Osteoma is a slow-growing tumor that generally enlarges in an affected area and does not cause any symptoms. The pathogenesis of osteoma remains unknown. Osteoma is classified into 3 types according to the site of origin. Central osteoma arises from the endosteum, peripheral osteoma originates from the periosteum, and extraskeletal osteoma develops within the muscle [1, 3].

The sites of osteoma development are usually restricted to the craniofacial bones and rarely include other bones. They occur most frequently in the mandible and paranasal sinuses. On rare occasions, osteomas had been found in the temporal bone [4], middle ear [5, 6], and tongue [7, 8]. Only 4 cases of osteoma of the larynx and 2 cases of the neck have been reported thus far [3, 9–13].

Excision of osteoma is often unnecessary; however, surgery is needed to reduce significant symptoms and to avoid dysfunction because of the growing tumor. We performed transoral resection to prevent dysphasia and respiratory distress.

To the best of our knowledge, the present report describes the first case of an osteoma in the pharyngeal space that developed from the hyoid bone.

## 2. Case Presentation

An 84-year-old man was referred to our hospital for evaluation of a pharyngeal tumor. His presenting symptom was a slight throat pain that developed 1 week before the consultation. He had no dyspnea or hoarseness. His past history included 4 years of chronic otitis media associated with dizziness.

**Figure 1:**
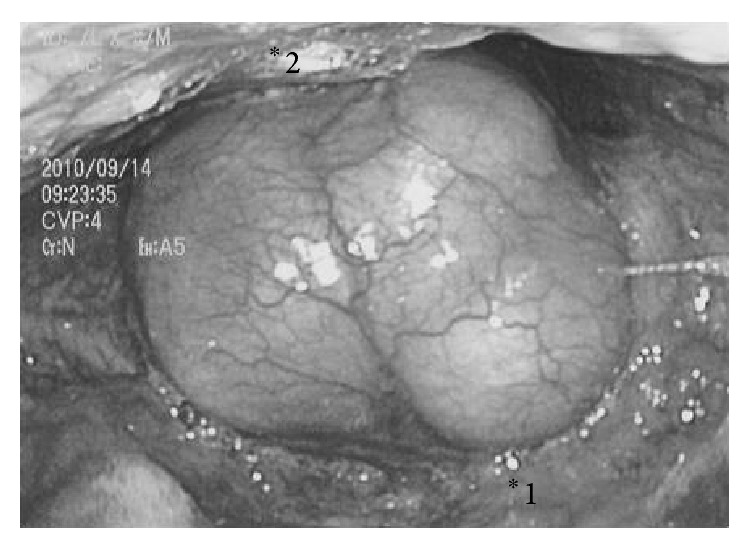
Endoscopic examination of the pharynx. ^*^1: root of the tongue; ^*^2: posterior wall of the pharynx.

**Figure 2:**
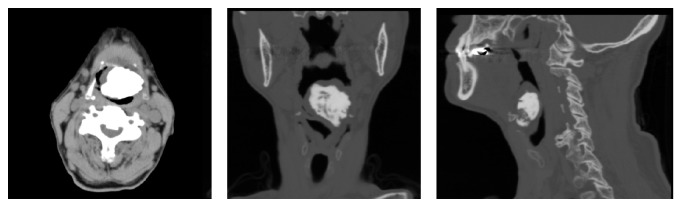
CT scan of the neck. A large osseous mass adjacent to the hyoid bone was observed.

Endoscopic examination revealed a huge mass occluding the pharyngeal space (Figure 1). The tumor had a smooth surface and was covered with normal mucosa. The epiglottis and vocal cord could not be confirmed. Caudally to the tumor, we could confirm normal vocal cords with normal mobility. A computed tomography (CT) of the neck showed a large osseous mass adjacent to the hyoid bone (Figure 2).

Transoral resection with tracheostomy was performed under general anesthesia. To orally observe the huge tumor, a mouth gag was used. The assistant kept the tongue retracted using a muscle hook. The tumor was covered with normal mucosa and was fixed at the left side of the epiglottic vallecula. We attempted to retract the tumor using a pick, but it moved only slightly. The left side of the tumor that was fixed to the vallecula area had to be blindly separated using scissors. This mobilized the tumor and allowed its removal from the surrounding tissue. There was only a small defect in the vallecula mucosa, which healed normally postoperatively. The bleeding was minimal, and there was no bleeding or swelling of the pharynx and epiglottis postoperatively. Two days after the operation, the tracheostomy was closed. Postoperative dysphasia or hoarseness was not observed.

**Figure 3:**
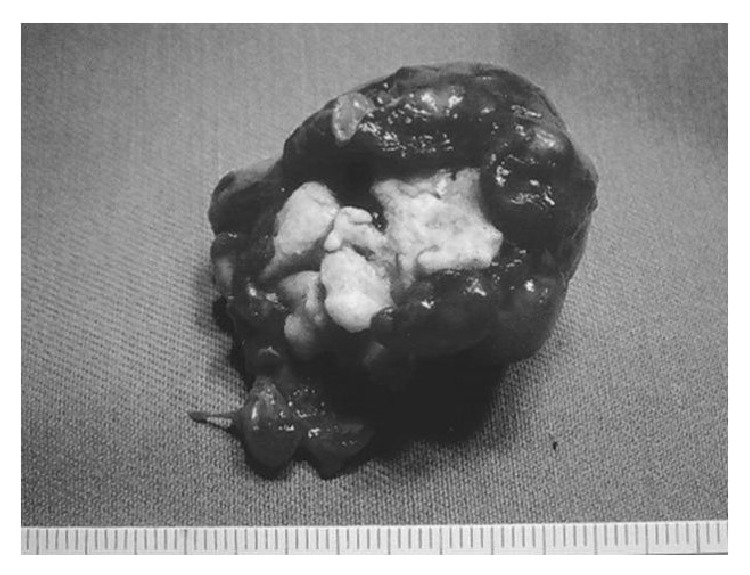
Tumor size and diameter. The tumor was round and 4 cm in diameter. An irregular osseous mass covered with mucosa was observed.

**Figure 4:**
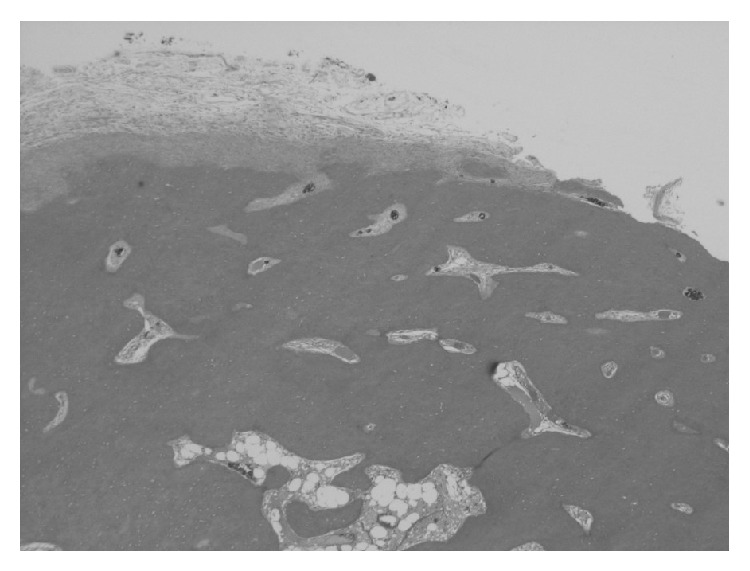
Histological examination of the tumor (H&E ×20). The tumor consisted of mature lamellar bone without a fibrous component. Bone marrow cells and trabeculae were confirmed in the center of the tumor.

The tumor was round and 4 cm in diameter. It was covered with normal mucosa (Figure 3). Histopathologically, the tumor consisted of mature lamellar bone without a fibrous component. Bone marrow cells and trabeculae were confirmed in the center of the tumor (Figure 4). On the basis of these findings, a diagnosis of osteoma of the pharynx was made.

For 2 postoperative years, the patient has been freefrom throat symptoms and signs of recurrence. However, 1 year postoperatively, his dizziness worsened prompting us to perform an operation for a cholesteatoma that destroyed the bony wall of the lateral semicircular canal.

## 3. Discussion

Most benign neoplasms that arise in the pharynx are papillomas, fibromas, hemangiomas, pleomorphic adenomas, and chondromas. Since these tumors grow slowly, surgical treatment is not always necessary. Masses that develop around the hyoid bone includethyroglossal duct cyst, aberrant goiter, and dermoid cyst. However, tumors that originate from the hyoid bone are rare. Only 14 cases of chondrosarcoma that develop from the hyoid bone have been reported in the English literature [14].

An osteoma presents as a protruding mass composed of abnormally dense but otherwise almost normal bone. Osteomas are formed by bones of osteoforming proliferation with membranous origin [15]. Osteomas are uncommon among head and neck tumors, but they commonly involve the skull. The most common site is the mandible (particularly the angle), followed by the nose and paranasal sinuses [16]. The frontal and ethmoidal sinuses are more frequently involved than the sphenoid and maxillary sinuses. Soft tissue osteoma, another type of osteoma, has also been reported. This tumor originates mainly in the posterior part of the tongue. About 40 cases of tongue osteomas have been reported [7, 8]. Cases of osteomas in the larynx (*n* = 4) or neck (*n* = 2) were rare (Table 1). All cases involved male patients, of whom 3 showed osteoma that developed from the cartilage in the laryngeal frames, 1 presented with coalescence of the stylohyoid process to the hyoid bone, and 2 showed osteoma that developed from soft tissue. Thus far, there has been no report on osteoma occurring in the pharyngeal space that developed from the hyoid bone.

Osteoma is formed by bones of osteoforming proliferation with membranous origin. Microscopically, it is slightly different from adult osseous tissue. Histologically, osteomas have 2 distinct variants [15]. One is the compact osteoma which is made of mature lamellar bones without harvest canals or a fibrous component. The other is trabecular osteoma which is composed of cancellous trabecular bone with marrow surrounded by a cortical bone margin. In the present case, the tumor consisted of mature lamellar bone with bone marrow cells and trabeculae without a fibrous component similar to the first type.

The pathogenesis of osteoma remains controversial. Osteomas can be divided into 3 types based on the site of origin, namely, central, peripheral, or extraskeletal type [1, 2]. Central osteomas arise from the endosteum, peripheral osteomas from the periosteum, and extraskeletal osteomas usually from within the muscle. Osteomas that develop within the muscle are called soft tissue osteomas. Various theories have been proposed to explain the pathogenesis of osteomas. One theory is that the lesions are true neoplasms. Another theory is that the lesions are associated with abnormal enlargement of embryonic tissues, previous traumas, or chronic inflammatory processes. According to Thoma and Goldman, growth starts spontaneously and is associated with trauma and not with inflammation [17]. Schneider et al. reported on 6 cases with a positive history of prior trauma [18]. In osteomas located in the muscle, pulling activities are suspected to develop the lesion. Varboncoeur et al. considered osteomas to be cartilage or periosteal embryonic remains [19]. However, a specific cause-effect relationship is difficult to establish.

A diagnosis of osteoma can be easily made by CT. These lesions are usually well-defined bony masses without enhancement effects. CT defines the full extent and the site of origin of an osteoma.

Most osteomas are usually asymptomatic. Although the excision of osteoma is often unnecessary, surgery is needed to reduce significant symptoms and to prevent dysphasia or dyspnea from an enlarged tumor. The type of procedure selected depends on the location or extent of the osteoma and the nature of any existing or anticipated complications. In the present case, the patient had a slight throat pain as symptom with advanced age but had no remarkable history. Since the mass was huge and occupied the laryngeal space, the larynx was not visible from the oral cavity. If the patient required an operation with general anesthesia, the anesthesiologist would not have been able to intubate orally. Therefore, we selected oral resection with tracheostomy to avoid dysfunction from a cervical approach. However, tumor removal was difficult as there was no useful instrument to manipulate the tumor in the vallecula. The use of only a mouth gag would not have enabled tumor observation. Moreover, the use of a laryngoscope would not have allowed us to handle the tumor since the operative field was narrow. For this reason, we used a mouth gag and a muscle hook which an assistant held in position perioperatively. There was no postoperative complication. For two years postoperatively, the patient has been free from any symptoms and signs of recurrence.

## 4. Summary

- This paper reports on apparently the first case of a pharyngeal osteoma that developed from the hyoid bone.
- Osteomas are benign, slow-growing tumors and are usually asymptomatic. Surgery is needed to reduce significant symptoms and to prevent dysfunction from an enlarged tumor.
- We performed oral resection with tracheostomy to avoid dysfunction from a cervical approach.

## Figures and Tables

**Table 1:** Cases of osteomas developed in larynx and neck.

Age	Sex	Site	Symptoms	Size	Originated site	Treatment
68	Male	Larynx	Hoarseness	2 cm	Cricoid cartilage	Excision
60	Male	Larynx	Incidental finding (difficulty intubation)	1.5 cm	Thyroid cartilage	Excision
79	Male	Larynx	Dysphonia	1.6 cm	Soft tissue	Excision
48	Male	Larynx	Dysphonia, dysphasia	N.A.	Arytenoid cartilage	Excision
63	Male	Deep neck	Incidental finding (neck CT)	2 cm	Stylohyoid chain	Observation
25	Male	Paravertebral space	Neck mass	6.5 cm	Soft tissue	Excision

Present case						
84	Male	Pharynx	Throat pain	4 cm	Hyoid bone	Excision

## References

[1] Woldenberg Y., Nash M., Bodner L. (2005). Peripheral osteoma of the maxillofacial region. Diagnosis and management: a study of 14 cases. *Medicina Oral, Patología Oral y Cirugía Bucal.*.

[2] Horikawa F. K., de Freitas R. R., Maciel F. A., Gonçalves A. J. (2012). Peripheral osteoma of the maxillofacial region: a study of 10 cases. *Brazilian Journal of Otorhinolaryngology*.

[3] Angelillo M., Mazzone S., Costa G., Mazzone A., Barillari U. (2009). The first case of osteoma in the false vocal fold. *Auris Nasus Larynx*.

[4] Ben-Yaakov A., Wohlgelernter J., Gross M. (2006). Osteoma of the lateral semicircular canal. *Acta Oto-Laryngologica*.

[5] Jang C. H., Cho Y. B. (2009). Osteoma of the incus with congenital cholesteatoma: a case report. *Auris Nasus Larynx*.

[6] Shimizu T., Okamoto K., Majima Y. (2003). Osteoma of the malleus: a case report and literature review. *The American Journal of Otolaryngology*.

[7] Nash M., Harrison T., Lin P.-T., Lucente F. E. (1989). Osteoma of the tongue. *Ear, Nose and Throat Journal*.

[8] Bernard P. J., Shugar J. M. A., Mitnick R., Som P. M., Meyer R. (1989). Lingual osteoma. *Archives of Otolaryngology: Head and Neck Surgery*.

[9] Batti J. S., Abramson A. (2000). First report of a case of osteoma of the larynx. *Ear, Nose and Throat Journal*.

[10] Redman A. G. O., Hide I. G., Zammit-Maempel I. (2000). Osteoma of the thyroid cartilage—an unusual cause of difficult intubation. *British Journal of Radiology*.

[11] Mehta R. P., Faquin W. C., Franco R. A. (2006). Pathology quiz case 1. *Archives of Otolaryngology—Head and Neck Surgery*.

[12] Weber A. L., Loewenheim H. M. (1993). Osteoma arising from the stylohyoid chain and manifesting as a neck and oropharyngeal mass. *Annals of Otology, Rhinology & Laryngology*.

[13] Li L., Wang Y., Zhao Y. F., Lu H. Z., Zou S. M., Luo D. H. (1993). Osteoma arising from the stylohyoid chain and manifesting as a neck and oropharyngeal mass. *Annals of Otology, Rhinology & Laryngology*.

[14] Saki N., Akhlagh S. N., Mostofi N. E., Ahmadi K. (2008). Chondrosarcoma of the hyoid bone: imaging, surgical, and histopathologic correlation. *Laryngoscope*.

[15] Nielsen G. P., Rosenberg A. E. (2007). Update on bone forming tumors of the head and neck. *Head and Neck Pathology*.

[16] Viswanatha B. (2012). Maxillary sinus osteoma: two cases and review of the literature. *Acta Otorhinolaryngologica Italica*.

[17] Thoma K. H., Goldman H. M. (1960). *Oral Pathology*.

[18] Schneider L. C., Dolinsky H. B., Grodjesk J. E. (1980). Solitary peripheral osteoma of the jaws: report of case and review of literature. *Journal of Oral Surgery*.

[19] Varboncoeur A. P., Vanbelois H. J., Bowen L. L. (1990). Osteoma of the maxillary sinus. *Journal of Oral and Maxillofacial Surgery*.

